# Exploring the effect of play on heart rate variability as a measure of positive emotional states in pigs

**DOI:** 10.3389/fvets.2025.1518153

**Published:** 2025-01-28

**Authors:** Karolína Steinerová, Annika Krause, Sarah E. Parker, Yolande M. Seddon

**Affiliations:** ^1^Department of Large Animal Clinical Sciences, Western College of Veterinary Medicine, University of Saskatchewan, Saskatoon, SK, Canada; ^2^Competence Area Behaviour and Husbandry, Research Institute for Farm Animal Biology (FBN), Dummerstorf, Germany

**Keywords:** play behaviour, pig, assessment of positive emotions, farm animals, positive animal welfare, heart rate variability (HRV)

## Abstract

Play behaviour has been suggested to be inherently rewarding for animals, inducing positive emotional states. The psychophysiological effect of emotions can be assessed through heart rate variability (HRV), serving as a proxy measure of sympathovagal balance. This study investigated how the performance of play influences heart rate (HR) and HRV parameters (RMSSD, SDNN) in pigs. It was hypothesized that HRV would increase during and immediately after play due to predominant vagal activation compared to baseline, indicating a positive emotional state. Gilts (*n* = 32, 18 and 19 weeks of age), housed in standard partly-slatted pens, were selected from two pen-level play treatments: Novelty (NOV) and Play Pen (PLP). Play treatment pigs were reared with intermittent play promotion (3x/week) from 10 weeks of age. For HRV recordings, play was promoted for 15-min in pairs of gilts within treatment, with destructible novel objects given either in the home pen (NOV, 1 m^2^/pig), or in an enclosed ‘playpen’ area providing extra space (PLP, 2.9 m^2^/ pig). HRV was measured during a play session in three consecutive periods: (i) baseline (before play bout, no play occurring), (ii) play bout (play expression), and (iii) after-play (immediately after play bout). Twenty-six gilts played at least once. Play bouts lasted between 10 and 30 s (10 s: *n* = 60, 20 s: *n* = 18, 30 s: *n* = 6). In 10-s bouts, compared to baseline, RMSSD was higher during play (*p* = 0.027) and after-play (*p* = 0.015), while SDNN increased during play (*p* ≤ 0.001) and after-play (*p* = 0.008) only with ambulation (pig moving forward: walking or running). HR did not differ across periods but was higher at ambulation (*p* = 0.003). Twenty-sec bouts followed the same relationship with only numerical differences, while HRV in 30-s bouts did not differ. Treatments did not influence HRV. Results suggest that engaging in play increases HRV, with this effect persisting into the period immediately after play. This indicates that play contributes to positive emotional states in pigs. Physical activity involved in play influences HRV. More dynamic and energetic play involving ambulation might be more rewarding for pigs. This study provides evidence for assessing positive emotions in pigs and underscores the importance of incorporating rewarding experiences into conventional farming practices.

## 1 Introduction

The promotion of positive experiences for animals in conventional farming practices has gained attention to improve welfare for farmed animals (1). Play behaviour is widely associated with pleasurable states in animals, as evidenced by behavioural observations (2, 3) and extensive neuropharmacological research on rewarding properties of social play in rats (4, 5). As a result, play has the potential to positively alter the animals’ affective state, including both emotions and longer lasting moods (6, 7). Based on this, the performance of play behaviour in farmed pigs has gained importance and has been investigated in relation to the improvements of housing conditions (8, 9), a proper development of social skills (10) and other behaviours such as feeding and drinking (11), and reaction to human handling (12). Rearing pigs with play opportunities has also been found to enhance their recovery after viral infection [disease resilience (13)]. This result aligns with the premise that the activated reward system and its associated release of neurotransmitters may act as a stress-buffer, alleviating the impact of stress (e.g., immunological stress during infection) on the organism [e.g., (14, 15)], and provides further evidence to suggest the performance of play confers positive emotions.

To gain insight in the emotional evaluation of pigs during play behaviour, the additional assessment of changes in the activity of the autonomic nervous system (ANS) is a promising physiological indicator of the emotional processing involved in different types of behaviour (16, 17). Heart rate variability (HRV) has been used as a proxy measure of the ANS in humans and animals, reflecting the rhythmic oscillation of cardiac activity (16). Emotional systems are distributed in the limbic forebrain that influences the ANS through controlling the activity of sympathetic and vagal tone at the heart, which affects sinoatrial node activity. As emotional perceptions can cause different shifts in autonomic balance, towards sympathetic or parasympathetic predominance, analysis of ANS activity is considered an appropriate approach to draw conclusions about changes in sympathovagal balance providing an insight into a psychophysiological influence of emotion regulation (1, 16, 18, 19). In particular, much attention has been paid to cardiac vagal tone as a psychophysiological marker of emotional regulation, which can be calculated from specific HRV parameters such as the root mean square of successive RR interval differences (RMSSD). High vagal tone is associated with more efficient autonomic control, which ensures increased sensitivity and reactivity of the organism to changes in the environment (20, 21).

Most scientific research on emotional states evaluated by HRV parameters has focused on responses to negative stimuli. For example, in the context of fear, a negative stimulus (electric shock) was associated with tachycardia accompanied by vagal withdrawal in rodents (22). This physiological reaction has also been reported in pigs in reaction to social stress (23), tail biting (24) and castration pain (25). These situations are thought to be characterized by a rather negative valence demonstrating a link between cardiac vagal tone and psychological components in negative contexts. Further, it has been suggested that the cardiac vagal tone is a potential indicator for positive emotions (1). This assumption is supported by studies demonstrating vagal activation in positive contexts. For example, in humans watching amusing videos, increased RMSSD indicated greater vagal activity, compared to fearful, neutral, and angry emotions (26). Similar reactions were found in pigs, showing a state of positive arousal when they were individually called to feeding in an operant conditioning paradigm (17). Nursery pigs that were regularly handled in a gentle manner showed a greater vagal activation during human-interaction test, indicating better adaptability and lower stress (27). A consistent finding across those studies is that positive emotions may increase vagal tone, whereas the opposite may occur with negative emotions. No study to date has investigated HRV in relation to play behaviour in pigs.

Nonetheless, the sudden bursts of movements during play increase the pigs’ physical activity, which has been shown to modulate HRV. In equine athletes, exercise reduced the standard deviation of normal sinus beats (SDNN), which represents the sympatho-vagal balance, as well as primarily vagally influenced RMSSD (28). A reduction in HRV during aerobic exercise has also been reported in humans (29). The impact of physical activity combined with a positive emotional component on HRV is currently unknown. To accurately assess HRV during play, the pig’s physical activity should be accounted for.

The objective of this study was to determine how the performance of play (play bouts) influences HR and HRV (SDNN, RMSSD) parameters in pigs. Combining assessment of the behavioural and physiological measures simultaneously during play, this work aims to provide new information on how play influences emotion in pigs. It was hypothesized that HRV would increase during and immediately after play bouts due to predominant vagal activation compared to baseline, indicating a positive emotional state.

## 2 Materials and methods

All experimental procedures were reviewed and approved by the University of Saskatchewan Animal Care and Use Committee, AUP protocol #20200022, in accordance with the guidelines of the Canadian Council on Animal Care. This study was conducted at Prairie Swine Centre (PSC), Saskatoon, Canada from January 2022 to March 2022.

### 2.1 Experimental set-up, animals, and housing

A subsample of 32 gilts (Large White × Landrace) was selected (8). The gilts were chosen from pigs reared within two pen-level treatments: NOV and PLP, collectively referred to as play treatments, that had been reared with regular play promotion since random allocation to the treatments at 10 weeks of age. For HRV analysis, at 18 and 19 weeks of age, 32 gilts were chosen from two batches (16 gilts/batch) from a total of 64 gilts from 16 pens (2 gilts/pen/treatment/batch selected) across two rooms. Half of the gilts were selected from each room, ensuring a balanced number of gilts per treatment (NOV or PLP) and batch. The selection was based on good health status (e.g., no ailments, such as hernias, wounds), no medical history and a behavioural reaction while fitting a HR monitor. All eligible gilts had the monitor fitted and pigs that remained still during the fitting process and appeared undisturbed by the belt on the chest were selected. Because more than 32 gilts met all the criteria, the final selection was made randomly from this pool of eligible gilts, ensuring that all selected gilts were balanced across treatments, pens, rooms, and batches and adhered to the aforementioned health and behavioural standards. Only gilts were considered for selection as sympathovagal activation towards a valenced stimulus might differ between genders [e.g., a greater increase in LF/HF in barrows when exposed to a novel situation, suggesting a greater stress susceptibility, (30)]. Moreover, by choosing only one gender, a more homogeneous group is created, which increases the ability to detect effects by limiting variability.

The detailed experimental set-up for treatments and play promotion is described in Steinerová et al. (8). Briefly, prior to the allocation to the treatments, pigs were raised in non-enriched conventional fully-slatted farrowing pens. At 3 days of age, pigs were tail-docked, and ear-notched for identification, and males were castrated. An analgesic (Metacam®, 0.4 mg/kg; Boehringer Ingelheim, ON, Canada) was administered via intramuscular injection to all pigs at the time of processing. At 4 weeks of age, pigs were weaned and moved to conventional fully-slatted nursery pens without any physical enrichment. Health checks were performed daily. After random treatment allocation at 10 weeks of age, pigs were housed in groups of eight, balanced for sex, in partially-slatted concrete pens (slatted area: 1.80 m × 1.95 m, solid concrete area: 2.30 m × 1.95 m), providing 1 m^2^ per pig. Each pen had one feeder providing feed ad libitum via auger with two feeding spaces per hopper and continuous access to water through one bite drinker. Pigs were fed a commercial pelleted grower feed (Grower RWA-Veggie, Masterfeeds LP, Humboldt, Canada) until 16 weeks of age, then switched to finisher feed (Finisher RWA-Veggie, Masterfeeds LP, Humboldt, Canada), meeting the nutrient requirements of swine published by the National Research Council (31). All pigs were ear-tagged for individual identification. A standardized point-source enrichment was installed to each experimental pen and consisted of a single piece of untreated spruce lumber (not renewed) with cotton rope (renewed weekly) hung at floor level (Supplementary Table 1).

Within the larger experiment (8), play was intermittently promoted in the NOV and PLP play treatments during a play session with novel objects provided in the home pen (NOV) or a specific playpen which also provided more space (PLP, 10 m × 2.35 m, 2.9 m^2^/pig, solid concrete floor) from 10 until 21 weeks of age. Play treatment pigs received two 30-min play sessions per day (10 am/1 pm), three days a week (Monday, Wednesday, Friday), totalling six play sessions per week. Novel objects consisted of six enrichment objects (cardboard, straw, plain popcorn, cotton rope, lumber, burlap; Supplementary Table 1) and rotated weekly in different combinations to reduce habituation. Each combination of the novel objects was provided consistently for one week, then rotated to the next combination the following week. On the third day of each week, the novel objects were sprayed with one of three types of diluted 100% pure essential oils shown to increase interaction with enrichment [garlic—(32); lavender—(33)] and activity [thyme—(34)]. The rationale behind the play promotion treatment is described in detail in Steinerová et al. (8). Briefly, larger space promoted play in piglets previously [e.g., (35)] and destructible objects sustain pigs’ interest for longer duration (36). Novelty, that supports play in pigs (37), was supported with intermittent play promotion, object rotation, and olfactory stimulation. Behavioural observations (8) showed that the extra space and the objects successfully promoted play until the finishing phase. Details about the enrichment objects are in Supplementary Table 1.

### 2.2 Heart rate variability measurement

#### 2.2.1 Equipment

A HR monitor (Polar H10, Polar Electro Oy, Kempele, Finland) with a 130 Hz sampling frequency, providing RR intervals stored in milliseconds, collected the data. The HR monitor transferred the RR intervals to a Bluetooth receiving device (iPhone 11, Apple Inc., CA, USA; tablet Samsung Tab Active Pro—SM-T547U, Samsung, South Korea) using the Elite HRV App (Elite HRV, Asheville, NC, USA). A maximum of two gilts were measured at the same time, each with its own monitor paired with a separate Bluetooth receiving device (phone or tablet). The monitor consisted of a small computer (approximately 5 cm × 3 cm) with a screen on one side and a sensor on the other, connected by a stretchy strip with electrodes embedded on one side and plain stretchy fabric on the other. To protect the monitor from manipulation by the pigs, a homemade Velcro belt was attached along the entire length of the monitor on the side with the screen, while leaving the side with the electrodes in contact with the pigs’ skin. Electrode gel (Spectra 360 Electrode Gel, salt-free, hypoallergenic; Parker Laboratories INC., NJ, USA) was applied to moisten the electrodes to improve conduction.

#### 2.2.2 Habituation and monitor fitting

Focal gilts were habituated to the HR monitor in their home pen with other pen mates present for two 15-min sessions, on two separate days 1 week before the HRV recording. Focal gilts were marked with blue spray (Raidex GmbH, Dettingen/Erms, Germany) at least 30 min before fitting the HR monitor and recording. Two experimenters entered the pen and fitted the belt on the gilt’s chest, applying electrode gel to the electrode strip while offering semi-sweet chocolate chips (Great Value, Walmart Inc., AR, USA) as a distraction. While fitting the belt, it was ensured that the electrodes made close contact with the pig’s skin, while the Velcro belt remained on the outer side. To ensure proper fit, the short side of the chest strap was positioned on the pig’s left side, with the electrode strip located over the area covering the heart. The belt was adjusted to avoid being too loose or too tight, preventing a loss of connection should the pig lay down. Gilts that did not stand still for fitting of the monitor were gently restrained in a corner using a pig board to facilitate fitting.

#### 2.2.3 HRV recording during play session

Before each HRV recording in each pen and monitor fitting, first non-focal animals were moved to an empty playpen in an adjacent hallway where they remained for the duration of the recording, leaving only focal gilts in the home pen.

HRV data were collected over a 15-min period simulating a play session between focal gilts, while the NOV treatment gilts were in the home pen and the PLP treatment gilts in the playpen. All types of novel enrichment objects (cardboard, straw, popcorn, cotton rope, lumber, burlap) were given in either the home pen or playpen to stimulate play behaviour. The enrichment objects were not sprayed with essential oils during the HRV recording.

Once the monitors had been fitted on both gilts (identical procedure as for habituation), the experimenters had left the pen and either thrown the novel objects to the home pen for the NOV treatment gilts or guided the PLP treatment gilts to a playpen (with novel objects prepared beforehand in the playpen) at the other end of the hallway, with at least 20 meters between the playpens with the pen mates and the focal PLP gilts. Immediately thereafter, the experimenters had hidden from the pigs’ sight, and HRV recordings began. There was at least 1 min between fitting and the onset of the HRV recording. The experimenters remained in the room (if recording NOV) or hallway (if recording PLP) for the entire recording period, monitoring the Bluetooth connection for any potential interruptions. If an interruption due to an improper fit of the belt and/or the lack of the gel on the electrode strips occurred, one experimenter entered the pen and re-adjusted the belt and re-applied the gel.

After the 15 min, the monitors were taken off from the gilts, the enrichment objects were removed from the pens, the PLP gilts were guided back to the home pen and the pen mates from the hallway were returned to their respective home pens.

#### 2.2.4 Data selection and correction

Lorex cameras (one camera/pen; 4 k Ultra HD IP Security Camera, Lorex Technology, Markham, ON, Canada) filmed the HRV recordings. Videos were rewatched and instances where pigs engaged in play behaviour (Table 1) for at least 10 s, referred to as play bouts (P), were recorded by one consistent experimenter who could not be blinded to treatments. Data was excluded when: play bouts occurred when the experimenter was in the pen; the belt was manipulated by a pen mate; the pig was lying down; feeding and manipulating a pen mate.

**Table 1:** Ethogram of locomotor, social and object play behaviour, in which focal gilts for HRV recording needed to engage in (one type or combination) for at least 10 s to be considered for a play bout.

Type of play behaviour	Description
Locomotor play	Solitarily performing excitable, and energetic body movements, such as (i) jumping or whirling around to face in a different direction on the spot (pivot); (ii) running forward (gambolling); (iii) quickly laterally displacing the head and neck in both horizontal and vertical planes (head-toss); (iv) dropping rapidly from an upright position to a sitting or lying position (flop); (v) rolling its entire body from one lateral side to the opposite lateral side while lying on its back (rolling).
Social play	Rough and tumble play, including mutual pushing, nudging, head knocking between two or more pigs using the snout, head, neck, and/or shoulders in an aroused/excited manner, or vigorously chasing another pig.
Object play	Engagement with an object by touching, chewing, shaking using the snout and/or mouth with/without carrying the object in the mouth in an aroused/excited manner, or moving, kicking the objects with the limbs in an aroused/excited manner.

Once a play bout was identified, a true baseline (TB) was selected. A true baseline i) had to occur within 2 min before the play bout, ii) be of the same duration as the play bout, and iii) be comparable to the play bout in terms of physical activity, such as involve the pig being active, standing on all fours in an upright posture (not lying down). When several such intervals were available, the time segment that was closest to the play bout was chosen to minimise changes or disturbances between baseline and play. If no time segment within the 2-min period before the play bout qualified as TB, a baseline chosen for the first play bout was used instead, termed a general baseline (GB). If a play bout did not have an associated baseline (either TB or GB), it was excluded from the analysis. An after-play period (AP), of the same duration as the play bout, started 1 s after the play bout finished, during which the pig could perform any behaviour and be in any posture. The type of play (Table 1) performed during a play bout was also recorded. Because play involves increased activity that influences HRV, whether the pig was moving forward (walking, running; intensity of activity not recorded), referred to as ambulation, was recorded in all periods (TB/GB, P, AP). The intensity of movements when the pig remained on the spot was not recorded. The following behaviours that could influence HRV, referred to as confounding behaviours, were recorded during baseline and after-play periods: i) eating popcorn: pig explores the floor and deliberately searches for popcorn, resulting in chewing and ingesting it, ii) exploring: pig sniffs, roots the floor and/or pen fixtures in a slow and steady manner, iii) excitement: pig explores the floor and/or pen fixtures with energetic movements, iv) lay down: pig shifts from standing on all fours to lateral and sternal recumbency.

Play bouts identified from the videos lasted between 10 and 30 s. The bouts of 10-, 20-, and 30-s durations were selected for subsequent artefacts and ectopic beats correction. Variance of HRV increases with the length of recording and comparing HRV measures of different durations is inappropriate (16, 18). Therefore, observed durations had to be standardized. To obtain a standardized dataset, play bouts with durations between 10 and 30 s were rounded to the nearest duration [e.g., a play bout from 05:30 to 05:43 (mm:ss) lasting 13 s was shortened to 05:33 to 05:43, resulting in a 10-s play bout]. The 10-, 20-, and 30-s bout durations were considered as a distinct category and compared only within the same duration.

To make the 10-s dataset more robust, the 20- and 30-s bouts were shortened to 10 s. Since the start of the after-play period could not be moved, to shorten the 20- and 30-s bouts to 10-s bouts, the first 10 (in case of 20-s) or 20 s (in case of 30-s) of the bout were removed. This ensured that the beginning of the after-play period remained the same [e.g., a play bout lasting 20 s from 11:20 to 11:40 (mm:ss) was shortened to 10 s from 11:30 to 11:40; after-play period started at 11:41]. The dataset with the 10-s bouts, including shortened 20- and 30-s bouts, represents the complete primary dataset. However, to explore HRV changes during and after play in longer play bouts, the original 20- and 30- s bouts were also preserved and analyzed separately.

Artefacts and ectopic beats (missed, extra and misaligned beat detections) in RR interval time series distort HRV analysis results (18, 38); therefore, all HRV recordings were corrected using the Kubios software (Kubios HRV Premium 3.4.1., Kubios Oy, Kuopio, Finland). The “automatic correction” method, previously validated for sensitivity and specificity (39), was used. The automatic correction identifies artefacts within a time series representing the differences between consecutive RR intervals. For detailed information, see the Kubios user guide (40). The recordings with more than 10% of corrected RR intervals were excluded (41, 42).

### 2.3 Statistical analysis

The RR intervals were analysed in the time domain, the simplest method to perform analysing the intervals between the successive heartbeats. Other types of analyses, such as frequency and nonlinear analyses require longer recordings of a minimum of 2 min (18, 43). HR and HRV parameters used for the analysis were calculated by the Kubios software and were as follows: the mean HR (beats per minute, bpm), the standard deviation of the RR of normal sinus beats (SDNN; ms), and the root mean square of successive differences between normal heartbeats (RMSSD; ms).

Statistical analysis was performed using STATA 17 software (StataCorp LLC, TX, USA). An underlying assumption for parametric testing was examined by assessing the normality of all data with the Shapiro–Wilk test. Based on the normality results and the distribution evaluated using histograms, the complete primary dataset of 10-s bouts (including shortened 20- and 30-s bouts) and the preserved original bouts of 20-s were analysed in a mixed effects linear regression model. The bout was the experimental unit. HR, SDNN and RMSSD were analysed in a separate model for each bout duration.

The clustering variables represented the hierarchical structure of the study (batch, room, pen, pig, bout), and their variability contributing to the dependent variables was assessed in an empty model. The proportion of total variance explained by random effects was assessed with the intraclass correlation coefficient (ICC). If the hierarchically nested clustering variables explained less than 5% of the variability, they were not included in the final models. When repeated observations within pig were taken (multiple play bouts per pig), the bout number was included in the random statement to account for similarities within pig. Models were built by forward stepwise inclusion of independent variables as fixed effects (*time period, treatment, ambulation, play type, confounding behaviour*). The *time period* (baseline, play bout, after-play) were included to explore HRV differences between baseline and play bout, as well as between baseline and after-play. The *treatment* was considered to investigate whether the two environments where play occurred made any difference on HRV. Physical activity influences HRV, which is why *ambulation* was included. Since pigs perform different types of play, the study also explored *play type*. *Confounding behaviour* was included to separate potential confounding effects of other behaviours (defined above) occurring during baseline and after-play periods. Because *play type* was not recorded in baseline and after-play period, and *confounding behaviour* was not recorded during a play bout, these two variables had to be assigned a value to avoid STATA treating them as missing. Therefore, *play type* was treated as a pig-level variable; baseline and after-play period were coded with the same play type as was recorded during the play bout (e.g., if a pig performed locomotor play in the play bout, locomotor play was also coded for baseline and after-play period). *Confounding behaviour* was treated as a lagged variable; a play bout was assigned the same value from the baseline (e.g., if exploration was recorded in baseline as a confounding behaviour, the same was coded in a play bout). If a variable was not significant during model building but caused a 20% change in effect for other variables, it was retained in the model and treated as a confounding variable. Interaction effects for significant variables only were assessed for inclusion, with three-way interactions included only if they remained significant alongside relevant two-way interactions in the model. Variables and their interaction effects were included in the final model if *p* ≤ 0.05 (Wald test). Model fit was evaluated using Akaike’s and Bayesian information criteria (AIC, BIC). Residuals of the final linear models were examined for normality and homoscedasticity.

The non-parametric Wilcoxon signed-rank test was used for analysis of HRV parameters with 30-s bout duration due to low sample size and because normal distribution of the data could not be assumed.

To explore how the performance of play influences HR, SDNN and RMSSD in pigs, the final models built according to the criteria mentioned above are shown in Table 2.

**Table 2:** Factors affecting measured effect of a play bout on heart rate (HR, beats per minute, bpm), the standard deviation of the RR of normal sinus beats (SDNN, ms), and the root mean square of successive differences between normal heartbeats (RMSSD, ms) in the complete primary dataset of 10-s (*n* = 84) bouts (including shortened 20- and 30-s bouts) and 20-s (*n* = 18) bouts: overview of the fixed effects, interactions and random effects included linear in the final multilevel multivariable regression models.

Bout duration	Outcome measures for separate models	Fixed effects					Final interaction	Random effects			Bout┼
		Time period	Treatment	Ambulation	Play type	Confounding behaviour		Room	Pen	Pig	
10 s	HR (bpm)	●		●						●	●
	SDNN (ms)	●		●			T^X^A			●	●
	RMSSD (ms)	●							●	●	●
20 s	HR (bpm)	●		●						●	●
	SDNN (ms)	●								●	●
	RMSSD (ms)	●						●	●	●	●

┼When repeated observations within pig were taken (multiple play bouts per pig), the bout number was included in the random statement to account for similarities within pig. T^X^A: the interaction between time period (T) ^X^ Ambulation (A). ● Signifies inclusion in the model.

To test whether the type of baseline (true/TB vs. general/GB) affected the output of the final models, a sub-dataset was created by excluding all periods within a bout that used GB (e.g., if play and after-play periods had GB as their baseline, these observations were excluded). The final model was then rerun, and the outputs compared. Regression model outputs with predicted means from models using only TB are reported in Supplementary Table 2.

Results are presented as predicted means of fixed or interaction effects and unadjusted lower and upper 95% confidence intervals (CIs). *p*-values are presented for the fixed effects. Significance thresholds (ST) were calculated using a Bonferroni correction (0.05/number of comparisons) to control overall *p*-values for analysis-wise error in multiple comparisons. Unadjusted p-values are presented for pairwise comparisons.

## 3 Results

### 3.1 Descriptive summary

From the initial sample size of 32 pigs, 30 pigs played at least once during the play session, with the total of 113 play bouts. Twenty-seven percent from the total number of play bouts exceeded 10% of ectopic beats and were excluded from the analysis. This resulted in 84 play bouts from 26 pigs (NOV: 12 pigs, PLP: 14 pigs), with 71% (*n* = 60) bouts lasting 10 s, 22% (*n* = 18) 20 s and 7% (*n* = 6) 30 s used for the analysis. The 20-s bouts were performed by 12 pigs (NOV: 4 pigs, PLP: 8 pigs) and the 30-s bouts by five NOV pigs. Pigs had between one to 10 play bouts per play session (NOV pigs range: 1–8, PLP pigs: 1–10; Figure 1). All pigs had their own baseline and play period within bout, but four pigs were missing an after-play period in one bout, one pig in two bouts, and one pig in four bouts. In the 10-s complete primary dataset, general baseline was used instead of true baseline in 42% of observations, and in the 20-s dataset in 38% of observations. Pigs engaged in object play in 77% of the play bouts, with the rest consisting of play type combinations: 5% object and social, 4% object and locomotor, 1% social and locomotor, and 12% all three play types. Ambulation was recorded in 77% of baseline, 42% of play, and 39% of after-play periods. Confounding behaviour was recorded in 83% of baseline and 82% of after-play periods (B: 10% popcorn, 20% excitement, and 54% exploration; AP: 3% popcorn, 9% excitement, 68% exploration, and 3% lay down).

**Figure 1:**
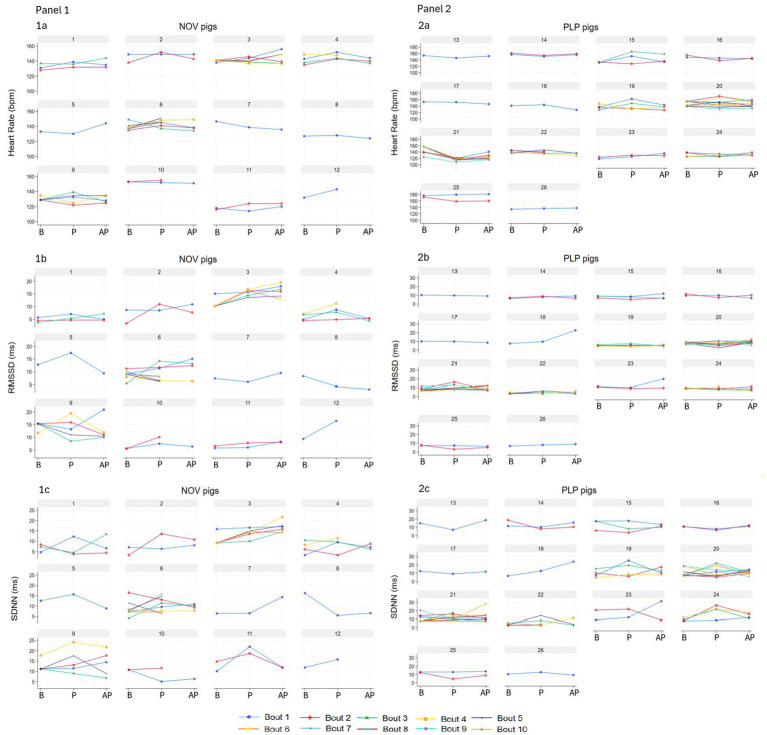
Heart rate (beats per minute, bpm, 1a, 2a) and its variability parameters (the standard deviation of the RR of normal sinus beats, SDNN, ms, 1b, 2b; the root mean square of successive differences between normal heartbeats, RMSSD, ms, 1c, 2c) of individual pigs in NOV (panel 1, *n* = 12, numbers 1–12) and PLP (panel 2, *n* = 14, numbers 13–26) treatments per individual 10-s bout during baseline (B, true and general), play (P) and after-play (AP) periods. Data are presented as observed.

### 3.2 Regression output

**Figure 2:**
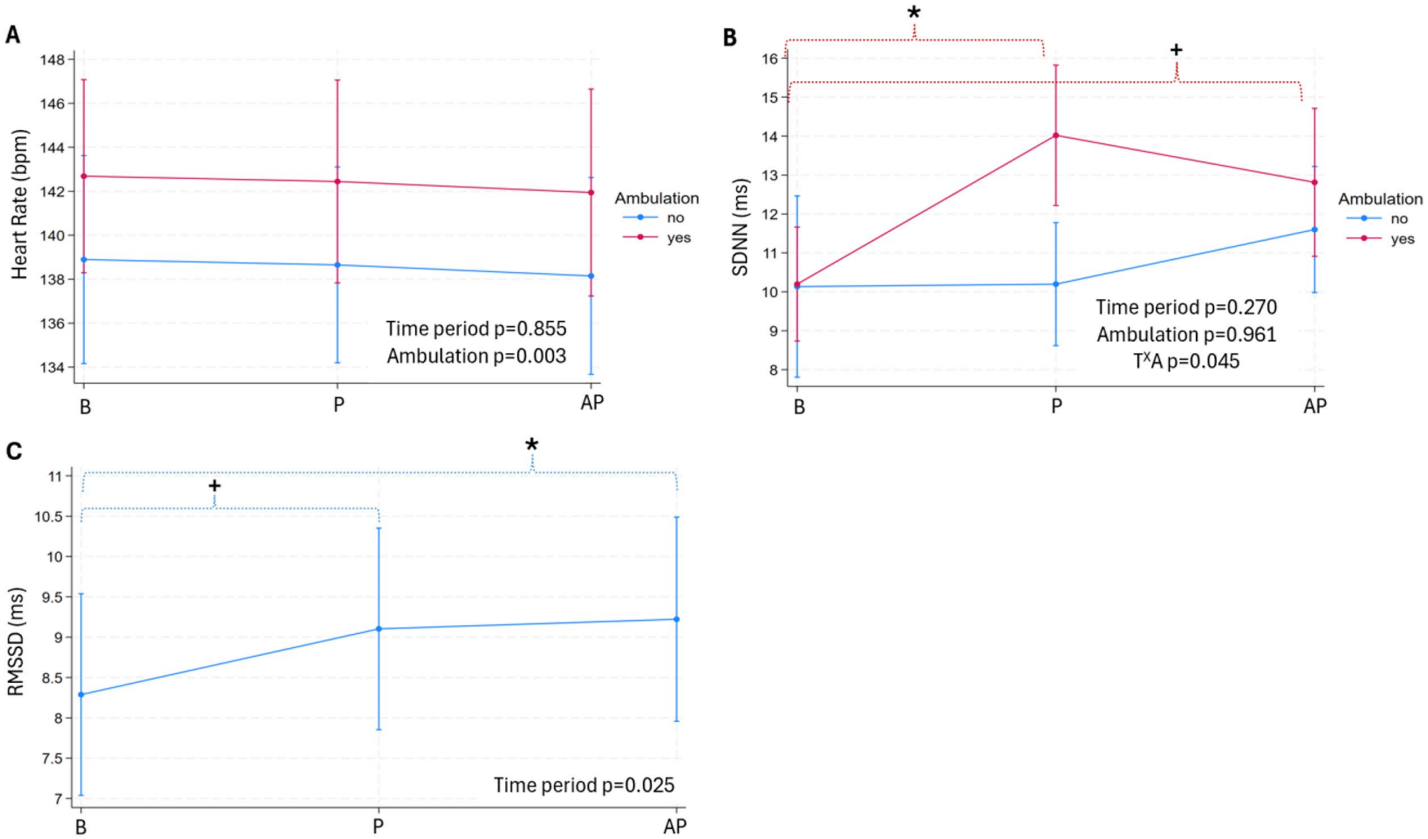
Heart rate (beats per minute, bpm, **(A)** and its variability parameters (the standard deviation of the RR of normal sinus beats, SDNN, ms, **(B)**; the root mean square of successive differences between normal heartbeats, RMSSD, ms, **(C)** per pig within 10-s bout (pigs: *n* = 26, bouts: *n* = 84) during baseline (B, true and general), play (P) and after-play (AP) periods. Data are presented as predicted means with 95% confidence intervals, with *p*-values of the fixed effects denoted on the graph. The intraclass correlation coefficient for random effects (ICC) are shown in the footnote. [Footnote: ‘X’ between two variables signifies an interaction effect. Significant differences, as defined by the significance threshold (ST) across time periods, are denoted on the graph with an asterisk (*) and above color-coded brackets. *p*-value for a pairwise comparison close to the ST is denoted with a plus sign (+). The ICC, p-values for pairwise comparison with the ST, type and number of comparisons (in italics and parentheses, respectively) are as follows: Graph A) ICC: pig: 0.60, bout: 0.63; B) ICC: pig: 0.22, bout: 0.22; the interaction between time period (T) ^X^ ambulation (A) – *ambulation yes/no within time period* (baseline yes/no: *p* = 0.961, play yes/no: *p* ≤ 0.001, after-play yes/no: *p* = 0.264); *ambulation yes/no across time periods*: baseline vs. play (A yes: *p* ≤ 0.001, A no: *p* = 0.960), baseline vs. after play (A yes: *p* = 0.008, A no: *p* = 0.248), ST (7): 0 = 0.007; brackets are color-coded to match the interaction term; C) ICC: pen: 0.14, pig: 0.55, bout: 0.55; the effect of time period—baseline vs. play (*p* = 0.027), baseline vs. after play (*p* = 0.015), ST (2): *p* = 0.025].

Results are presented as predicted means and 95% CIs. The significance thresholds (ST) of the Bonferroni correction are reported in the text and footnote of Figure 2 as: ST (number of comparisons): the threshold’s *p*-value’ in *italics*. Results are reported from regression models including both types of baselines (TB and GB) unless otherwise stated.

The HR during play and after-play periods did not differ from baseline in the 10-s (Figure 2A), 20-s, and 30-s bouts (Table 3). Ambulation influenced HR in the 10-s and 20-s bouts, with HR being faster in the 10-s bout (Figure 2A), and the 20-s bout when pigs were moving forward (ambulation yes: 148.38 [140.73, 156.03]; ambulation no: 142.01 [134.20, 149.83], *p* = 0.028; the predicted mean [95% CIs], bpm).

**Table 3:** Heart rate (HR, beats per minute, bpm) and its variability parameters (the standard deviation of the RR of normal sinus beats, SDNN, ms; the root mean square of successive differences between normal heartbeats, RMSSD, ms) per pig within 20- (pigs: *n* = 12, bouts: *n* = 18) and 30-s (pigs: *n* = 5, bouts: *n* = 6) bouts during baseline (B, true and general), play (P) and after-play (AP) periods as provided by separate multilevel multivariable regression models (20-s bouts) and Wilcoxon signed-rank test (30-s bouts).

Bout duration and statistical analysis type	Parameter	P-value		20 s: predicted mean [95% CIs]; 30 s: observed mean [min, max]; sec			AIC	ICC
		Time period	Ambulation	Baseline (B)	Play (P)	After-play (AP)		
20 s, Mixed Linear Regression								
HR (bpm)		0.387	0.028	147.64 [139.66, 155.62]	143.90 [136.14, 151.65]	144.41 [136.57, 152.26]	416.42	pig: 0.71; bout: 0.71
+SDNN (ms)		0.104	-	11.88 [9.65, 14.12]	11.93 [9.69, 14.16]	14.88 [12.64, 17.11]	333.54	pig: 3.93E-10; bout: 3.93E-10
+RMSSD (ms)		0.230	-	7.98 [5.50, 10.46]	8.41 [5.93, 10.89]	9.07 [6.59, 11.55]	256.22	room: 0.53; pen: 0.62; pig: 0.62; bout: 0.62
30 s, Wilcoxon Signed-Rank Test*								
HR (bpm)		B vs. P: 0.250, B vs. AP: 0.250	-	139.83 [134.00, 149.00]	142.67 [131.00, 147.00]	145.25 [139.00, 156.00]	-	-
SDNN (ms)		B vs. P: 0.094, B vs. AP: 0.125	-	9.33 [6.20, 12. 30]	13.60 [6.40, 18.10]	14.3 [10.10, 18.90]	-	-
RMSSD (ms)		B vs. P: 0.313, B vs. AP: 0.375	-	10.35 [6.20, 14.30]	12.28 [7.40, 16.70]	10.98 [8.60, 15.00]	-	-

For the 20-s bouts, the model output presented includes the p-value of period, and ambulation where applicable, along with pairwise comparisons and their threshold of significance, predicted means with 95% confidence intervals (CIs), model fit (Akaike information criterion; AIC) and the intraclass correlation coefficient for random effects (ICC). For the 30-s bouts, the p-values from each test analysing differences between B vs. P, and B vs. AP, along with observed means* and their minimum and maximum values, are reported. A hyphen (−) in the cell denotes not applicable (for linear regression: the fixed effect not significant during model building, and thus was not included in the model; for Wilcoxon test: could not be evaluated). For Wilcoxon Rank-Sign Test: exact p-values are reported. *Although the Wilcoxon signed-rank test evaluates the null hypothesis (H₀) that the median of the differences between the two paired samples (B vs. P, B vs. AP) is zero, the observed means are presented in the table instead of medians for consistency. +Although the SDNN and RMSSD in the 20-s bouts did not meet the threshold of significance, there was a numerical difference, with after-play period being higher compared to baseline (SDNN: 3.00 [−0.16, 6.16], *p* = 0.063, ST (2): *p* = 0.025, RMSSD: 1.09 [−0.17, 2.35], *p* = 0.089, ST (2): *p* = 0.025; the contrast of predicted mean [95% CIs], ms).

The SDNN and RMSSD during play and after-play periods differed from baseline in the 10-s bouts (Figures 2B,C), but not in the 20- and 30-s bouts (Table 3). For SDNN, there was a significant interaction between time period and ambulation (Figure 2B). Compared to baseline, SDNN was higher during both play (*p* ≤ 0.001, ST (7): *p* = 0.007, Figure 2B) and after-play (*p* = 0.008, ST (7): *p* = 0.007, Figure 2B) periods when pigs engaged in ambulation. In contrast, there was no difference in SDNN across periods when pigs were not moving forward (no ambulation, Figure 2B). SDNN was higher during play bout when ambulation was recorded compared to play bout when pigs stayed on the spot (*p* ≤ 0.001, ST (7): *p* = 0.007, Figure 2B), but this difference was not seen between baseline and after-play periods. In the 10-s bouts, RMSSD was elevated during play (*p* = 0.027, ST (2): *p* = 0.025, Figure 2C) and after-play (*p* = 0.015, ST (2): *p* = 0.025, Figure 2C) periods compared to baseline.

During model building, treatment was not a significant fixed effect and thus, was not included in the regression models.

When play and after-play periods were compared to TB only, HR did not differ (*p* ≥ 0.05), aligned with observations including both baselines. Regarding the influence of ambulation on HR during the 10- and 20-s bouts, the direction of the effect remained the same as in the analysis using both baselines; however, the difference was no longer significant. Similarly, for SDNN and RMSSD in the 10-s bouts, the relationship remained, but the *p*-values indicated only a tendency towards significance (SDNN: *p* = 0.059, interaction between time period and ambulation; RMSSD: *p* = 0.068, effect of time period). In the 20-s bouts, the time period did not influence SDNN and RMSSD (p ≥ 0.05), consistent with observations that included both baselines (Supplementary Table 2).

## 4 Discussion

This study explored the autonomic response to play bouts in pigs using HR and HRV parameters. Based on previous research indicating that play is inherently rewarding for animals (2, 44, 45), it was hypothesized that HRV would be higher during and immediately after play bouts compared to baseline, reflecting a positive emotional state. The current findings suggest that the performance of play increases HRV; however, physical activity, performed during and immediately after play, influences HRV, which is an important factor to consider while interpreting the results.

Generally, an increase in the parasympathetic activity, resulting in higher HRV, has been associated with positive emotions and emotional states (46). In the current study, SDNN and RMSSD, were elevated when pigs engaged in play behaviour and remained elevated during the immediate period following play. However, for SDNN, this was the case only when the pigs were moving forward (walking/running; ambulation), whereas ambulation did not influence RMSSD. Both parameters reflect different aspect of the ANS; SDNN reflect the long-term cardiac activity and is sympatho-vagally mediated, while RMSSD indicates the high frequency beat-to-beat variations and represents the vagal regulatory changes (1, 43, 47). To what extent the sympathetic and vagal systems influence SDNN is not known; and several interpretations have been proposed. Some authors consider SDNN to be predominantly sympathetically regulated [e.g., (48)], while others argue that the vagal tone plays a major role (49, 50). Moreover, generally understood reciprocal activation of both systems with antagonistic effects (one goes up, the other goes down) has been also challenged. It has been proposed that a dually innervated heart is affected by the uncorrelated sympathetic and parasympathetic (vagal) axes (51). In the current study, it was attempted to account for the effect of physical activity on HRV by choosing baseline with similar level of physical activity as during a play bout. Despite this attempt, the interpretation of the current SDNN findings, where the value increases during play and after play only with ambulation, remains challenging.

Play behaviour is characterized by energetic and excitable movements (2), either involving only the head (e.g., head knocks, shaking an object) while the pig remains on the spot, or by moving the whole body forward (e.g., galloping). The increased oxygen demand during aerobic exercise activates the sympathetic system and reduces parasympathetic activity (29). This, in turn, increases HR and thereby decreases HRV (29, 52, 53). For example, HR of exercising horses was significantly higher than during rest, with a progressive increase in beats per minute as exercise intensity increased from walking to trotting (54). Their SDNN and high-frequency (HF) component of the spectral analysis representing vagal tone decreased, whereas low frequency (LF) component (sympathetic activation) increased (54). Li et al. (28), also reported lower HR, SDNN and RMSSD during race training of equine athletes compared to rest. Although no changes were seen in the HF and LF peaks, nonlinear SD1 (Poincaré plot standard deviation (SD) perpendicular the line of identity), correlating with HF and RMSSD, and SD2 (Poincaré plot SD along the line of identity), correlating with LF power, were also decreased by exercise (28). Although the level of physical activity of horses is substantially more intensive than the physical activity of pigs during or after play in the current study, the equine findings illustrate the relationship between exercise, HR and HRV applicable to other mammals. Aligning with the equine studies, in the current study, HR was higher when the pigs were at motion compared to staying on the spot; but importantly, no change was observed during play and immediately after play compared to baseline. Speculatively, the rise in SDNN during play and after play only during ambulation, could involve the activation of both the sympathetic and vagal systems, representing non-reciprocal concurrent increase. At this stage, it is unknown to what extent each branch influenced SDNN increase.

Although the findings support the view that ANS activity is strongly influenced by physical activity (28, 29, 54), the higher RMSSD during play and after play compared to baseline, also suggest that the engagement in play was positively valenced. Therefore, if physical activity involves an emotional component, such as fun, joy, and pleasure, it is plausible that the vagal tone is co-activated, as suggested by the findings in this study. Furthermore, if the branches were reciprocally influenced, an increase in RMSSD (vagal activation) would result in reduced HR (sympathetic withdrawal). This was not seen in the current findings, where HR was higher only during ambulation, regardless of whether the pig played or not. However, the activation of the sympathetic branch resulting in a higher HR can be an indicator of arousal that occurred during ambulation. This further suggests co-activation of both systems: the sympathetic branch indicating arousal, and the vagal tone suggesting positive valence. Consequently, it can be speculated that more dynamic play involving ambulation might be more rewarding for pigs, due to greater arousal.

Similarly to the current study, pigs reared with cognitive enrichment, intending to provide a positive stimulus in their housing environment, demonstrated autonomic responses associated with positive emotions (17, 42). Through classical operant conditioning, pigs learned to associate an individual acoustic signal with receiving a feed reward at the feeder, and when called, they began to anticipate the reward, resulting in higher SDNN, RMSSD, and HR. (17, 42) Krause et al. (55), also reported sympathetic and vagal activation in pigs when they anticipated food. Anticipation of a positive event might have aroused the pigs and increase their SDNN, and, HR as well as stimulate the vagal tone and increase their RMSSD, indicating a positive emotional state (42). Access to food, as a motivating and positively appraised stimulus, aligns with assumptions about play behaviour, likely eliciting a similar, but perhaps more pronounced, cardiac response. Nevertheless, as observed in the current study, the increase in RMSSD during play and after-play, though significant, did not exceed 1 ms compared to the baseline RMSSD, which is lower than what has been reported in the aforementioned studies [(55): absolute difference of 5 to 13 ms; (17, 42): 2 ms]. Possibly, this indicates that the increase in RMSSD represents a positive response, albeit less intense than the response triggered by a feeding stimulus. Considering that HR did not change between baseline and play, the current findings indicate that such a small difference in the vagally influenced RMSSD was sufficient to prevent an increase in the sympathetically mediated HR.

Since play is an arousing and energetic activity, it can be suggested that the effects induced by play would remain even after the pig stopped playing. Supporting this assumption, SDNN and RMSSD values during play and after-play periods did not differ and were higher than those in baseline. A previous study reported that grow-finish pigs had elevated cortisol levels, suggesting increased arousal, following a 30-min play session in a standard production pen (8). The findings suggest that the physiological effects of play continued to be present also during post-play period which might prolong the rewarding feelings and ultimately enhance pig welfare.

The current study demonstrated that using HRV to assess whether play contributes to positive emotional states are confounded with physical activity involved in play. Namely, SDNN and HR that are (partly or fully, respectively) influenced by the sympathetic branch, are modulated according to the physical activity exerted during a play bout. Play is a dynamic behaviour, taking various forms, for instance, it can be a solitary object play, locomotory energetic pivots on the spot, mutual social chasing or solitary scampering, using the available space (56). A major limitation of this study is that the intensity of play exhibited when a pig played on the spot was not accounted for (e.g., vigorously shaking enrichment vs. moderate excitement and manipulation of enrichment). Some level of intensity happening on the spot was addressed in the variable *type of play* (locomotor, social, object), since each play requires a different level of physical involvement. For instance, locomotor play would be the most energetically demanding, most often involving forward motion of the whole body usually at a higher speed. On the contrary, object play mostly involves only the movement of the front body parts with or without ambulation, making it less physically demanding. However, even locomotor play can happen on the spot, and object play may involve ambulation, making this rationale not always plausible. Regression models were built using a stepwise forward inclusion of the variables. The variables were added one by one based on the objective of the study and relevance identified in previous literature, starting with time period (baseline, play, after-play), followed by type of play or ambulation. Although type of play was significant in the model with period, when the variable ambulation was added, the play type was no longer significant and thus was excluded from the regression (ambulation always stayed significant during the model building process). Based on this, it can be preliminarily concluded that forward motion had a greater effect on HRV measures than intensity of the physical activity involved in different types of play. Certainly, the effect of physical activity exhibited during play, including its type and intensity, whether stationary or in motion, should be considered in future studies investigating the autonomic response to rewarding and arousing behaviours, such as play.

Regarding a baseline measurement in the current study, considering that HRV was sampled during multiple play bouts within the 15-min play session, the baseline may have been influenced by prior autonomic fluctuations. To control for this, when possible, each play bout was given its own “true baseline (TB),” measured within 2 min before the play bout. This approach aimed to standardize data collection by focusing on the absolute change between the TB and the play or after-play periods. However, this current approach could also represent a limitation of the study that either magnified or reduced the absolute differences. Another approach would be to use a single baseline for all play bouts, resembling the general baseline (GB) used in the current study, when no TB was available. In fact, because some play bouts happened in quick succession, or the pig engaged in behaviours that excluded the possibility of using the time segment happening within 2 min before the play bout as TB (e.g., manipulation of a pen mate, lying down), approximately 40% of observations from 10- and 20-s bouts, used GB instead.

It can be argued that the use of GB in place of TB might introduce bias or variability, for example, by neglecting the impact of instantaneous changes in parasympathetic activation on HRV parameters. This possibility was explored through an analysis using only results where a TB was available. Although excluding observations with GB reduced the sample size and the power of the analysis, it was considered a necessary robustness check for the validity of the presented results. In contrast to the dataset with both baselines, ambulation no longer had a significant effect on HR. Nevertheless, the direction of the effect remained the same, with a smaller difference in measures. It may be that the effect is overestimated when baselines that are not true to the actual period are included. However, the smaller sample size may also mean that this altered effect is due to chance. But because the results were similar, any effects from bias or increased variability are likely to be minimal. Considering that TB is more representative of a baseline state before play, a dataset using TB only would be preferred. Other studies employed various methods to obtain baseline, and standardized methods are lacking, for instance, Zebunke et al. (17), averaged the values of time intervals from −60 to −20 s before an event of interest for baseline, and Luna et al. (57), used a 17-min baseline HRV measurement period.

Inclusion of the gilts that were calm and neutral to the equipment could have introduced bias to the study. The coping style of how the pig adapts to environmental challenges, in the current case, human handling and equipment fitting, may have influenced the autonomic reaction (55). Arguably, the current study might have unintentionally selected gilts that would classify as reactive or low-resisting (determined in a standardized behavioural back test), that showed lower HR during feeding, resting and handling, compared to proactive pigs (55). However, the rationale to select calmer gilts was to avoid any distressing restraint techniques (e.g., snaring) that affects HR (58) and could potentially outweigh the effects of play. If there was a personality effect, speculatively, because only calm animals were used, that effect would be the same for all gilts, not confounding the results. Using only gilts may also limit generalizability of findings to all pigs, including barrows. The non-invasive data collection method compromised the quality of the data, leading to the exclusion of 27% of it during data cleaning. This could be omitted by using an implanted telemetric device as in Krause et al. (55, 59); however, surgical implantation was not an option in the current study. This experiment was conducted in a production barn with pigs scheduled for slaughter, aiming to replicate a real-world scenario regarding the effects of play in a conventional production environment.

The nature of play bouts in pigs, occurring suddenly in short bursts, yielded short recordings of 10 to 30 s. Time segments of 10 s for HRV analysis in the time domain in pigs was also used in Zebunke et al. (42). As concluded from human populations, where a continuous, undisturbed recording is possible, it is recommended to have at least 5-min time segment to reliably estimate the parameters (18). Several authors aimed to validate this claim by correlating 5-min recordings with shorter segments. For example, HR could be reliably estimated from 10 s of RR intervals, RMSSD from 30 s (60, 61), and SDNN from 240 s (60). The short recordings also prevented frequency domain analysis that requires a minimum of 2 min of continuous data (18). This was not feasible in the current experimental setup, primarily because the pigs were measured in pairs and interacted with each other’s HR monitors. However, the parameters of the frequency domain analysis provide analogue information to those from the time domain analysis and RMSSD was described as more suitable and robust concerning the length of time segments to be analysed (68). For instance, the HF component of the frequency spectral analyses, which is reflected in respiratory sinus arrythmia, indicates the vagal input of the heart and correlates strongly with RMSSD (18, 62). Non-linear metrics would be a valuable addition outlining the unpredictability and complexity of HRV. However, Shaffer et al. (63), determined that for a visual analysis for patterns using a Poincaré plot and its measurements (SD1, and SD2), at least 90 s of recording is required. Nonetheless, both RMSSD and SD1 represent short-term HRV; and SD2 correlates with LF power, resulting in a closer relationship with SDNN (43). Moreover, since this is the first trial investigating the relationship between HRV and play, the simplest HRV analysis using the time-domain was considered sufficient to achieve the objectives.

Whether play was promoted in the home pen (NOV treatment), or in the larger playpen (PLP treatment) did not have any effect on HRV measures. This is a valuable finding. If play has the potential to improve the quality of life of farmed pigs, is positive for pigs and benefits their resilience against common production challenges (8, 13, 64), then having evidence that its rewarding properties are equal in both environments adds another incentive to incorporate the promotion of play into standard farming practices, regardless of the setting.

In summary, the findings indicate co-activation of the sympathetic and parasympathetic branches in the ANS during the performance of play and immediately after play, as evidenced by the increase in SDNN and RMSSD. The engagement of play increased the vagal tone, suggesting positive emotional states. This could have wider beneficial implications for pigs, as positive emotional states have been associated with better health in humans (65, 66) and in farm animals (67). This premise was supported by a study in the series on play behaviour in pigs by Steinerová et al. (13), which showed that pigs reared with regular play opportunities were less affected by Porcine Reproductive and Respiratory Syndrome Virus and demonstrated better performance during the infection, suggesting enhanced disease resilience.

Lastly, play involves physical activity that modulates the cardiac response, making the interpretation challenging. However, this study can be considered a preliminary step towards investigating vagal activation in a play context. To differentiate the effects of play on HRV from those of the physical activity itself, future studies should record the type and intensity of physical activity during play. Focus should also be placed on collecting longer time segments in more subjects, allowing for the expansion of HRV analysis from the time domain to the frequency domain, as well as non-linear analysis. Longer time segments would also facilitate the selection of a more appropriate baseline, enabling the autonomic response to return to its pre-event resting state after any arousing events.

## 5 Conclusion

The results suggest that the performance of play increases HRV, contributing to positive emotional states in pigs. These effects persist into the immediate period following play, indicating that the rewarding feelings extend beyond the active performance of play. The physical activity performed during play influences HRV, and dynamic play involving ambulation might be more rewarding for pigs. This study highlights the necessity of incorporating rewarding experiences into conventional farming practices.

## Data Availability

The raw data supporting the conclusions of this article will be made available by the authors, without undue reservation.
