# Supplementary material for: Exploring the effect of play on heart rate variability as a measure of positive emotional states in pigs
Source: Front Vet Sci. 2025 Jan 28;12:1518153. doi: 10.3389/fvets.2025.1518153 (PMC11812062; doi:10.3389/fvets.2025.1518153)
Supplement: Supplementary file 1 [file Table_1.DOCX]

Supplementary material

Table 1. The description and manufacturer of novel objects and essential oils for play promotion and standard point-source enrichment provided in Novelty (NOV) and Play pen (PLP) treatments. Adapted from (Steinerová et al., 2024).

| Novel  object for play promotion | Description | Manufacturer |
| --- | --- | --- |
| Cardboard | Three pieces of cardboard cut in 0.6 m x 0.2 m rectangles. | Uline corrugated boxes, Pleasant Prairie, WI, USA |
| Straw | Two to four litres of straw (incrementally increased weekly). | Simply Straw, fine cut 100% wheat straw, Lacombe, AB, Canada |
| Popcorn | Plain popcorn popped from 40 g of raw kernels at the facility. | Original kernel popcorn, Orville Redenbacher Popcorn, Chicago, IL, USA |
| Cotton rope (+ chain in NOV) | Unravelled three-strand cotton rope. For NOV: one 1.2 m strand of the rope tied to a 0.7 m chain with a carabiner. For PLP: two 0.7 m strands of rope/play pen. | Ropeshop.ca, Hamilton, ON, Canada |
| Lumber + chain | Two composites of a 2-piece untreated spruce lumber cut in 0.3 m x 0.1 m on a 0.3 m chain. | The Home Depot Canada Inc., Toronto, ON, Canada |
| Burlap | Two 0.3 m strands of burlap on the same chain as lumber. | Uline burlap roll, Pleasant Prairie, WI, USA |
| Essential oils | | |
| Garlic | 100 % pure essential oil diluted in 15 drops of oil/litre of water. | Divine Essence, Union Nature Aroma-Phyto Inc., Mt-Royal, QC, Canada |
| White thyme | 100 % pure essential oil diluted in 30 drops of oil/litre of water. | Aromaforce, Bioforce Canada Inc., Brunswick, QC, Canada |
| Lavender | 100 % pure essential oil diluted in 30 drops of oil/litre of water. | Naka Platinum, Naka Herbs and Vitamins, Toronto, ON, Canada |
| Standard point-source enrichment (all experimental pens) | | |
| Lumber + cotton rope + chain | A single piece of untreated spruce lumber cut in 0.6 m x 0.1 m with 1.2 m unravelled three-strand, ½-inch diameter cotton rope tied to a chain with a carabiner at floor level. | Lumber, chain: The Home Depot Canada Inc., Toronto, ON, Canada; Rope: Ropeshop.ca, Hamilton, ON, Canada |

Reference:

Steinerová, K., Parker, S. E., Brown, J. A., & Seddon, Y. M. (2024). The promotion of play behaviour in grow-finish pigs: The relationship between behaviours indicating positive experience and physiological measures. *Applied Animal Behaviour Science*, *275*, 106263. https://doi.org/10.1016/j.applanim.2024.106263

Table 2. Regression model output for heart rate (HR, beats per minute, bpm) and its variability parameters (the standard deviation of the RR of normal sinus beats, SDNN, ms; the root mean square of successive differences between normal heartbeats, RMSSD, ms) per pig within 10- (pigs: n = 23, bouts: n = 49) and 20-sec (pigs: n = 8, bouts: n = 11) bouts during true baseline, play and after-play periods as provided by separate multilevel multivariable regression models. The model output presented includes the p-value of time period, ambulation and its interaction where applicable, model fit (Akaike information criterion; AIC) and the intraclass correlation coefficient for random effects (ICC).

| Bout duration | Parameter | P-value | | | AIC | ICC |
| --- | --- | --- | --- | --- | --- | --- |
|  |  | Time period (T) | Ambulation (A) | Interaction: T^X^A |  |  |
| 10 sec |  |  |  |  |  |  |
|  | HR | 0.971 | 0.142 | - | 1029.81 | pig: 0.64; bout: 0.72 |
|  | SDNN | 0.167 | 0.893 | 0.059 | 849.92 | pig: 0.32; bout: 0.32 |
|  | RMSSD | 0.068 | - | - | 705.72 | pen: 0.12; pig: 0.58; bout: 0.58 |
| 20 sec |  |  |  |  |  |  |
|  | HR | 0.188 | 0.325 | - | 248.92 | pig: 0.79; bout: 0.84 |
|  | SDNN | 0.289 | - | - | 217.52 | - |
|  | RMSSD | 0.103 | - | - | 146.18 | - |

A hyphen (-) in the cell of p-values indicates that the fixed effect was not significant during model building, and thus was not included in the model.

Predicted means with 95% unadjusted confidence intervals from the model output in supplementary Table 2.:

**10 sec**

- HR (bpm)
  - Time period
    - Baseline: 140.2 [135.4, 145.09]
    - Play: 140.0 [135.2, 144.8]
    - After play: 139.9 [135.0, 144.8]
  - Ambulation
    - Ambulation yes: 141.05 [136.3, 145.8]
    - Ambulation no: 138.88 [134.1, 143.7]
- SDNN (ms)
  - Time period x Ambulation interaction
    - Ambulation no#Baseline: 10.8 [7.8, 13.7]
    - Ambulation no#Play: 9.9 [7.8, 12.1]
    - Ambulation no#After play: 12.3 [10.1, 14.6]
    - Ambulation yes#Baseline: 11.0 [9.0, 13.0]
    - Ambulation yes#Play: 14.4 [12.0, 16.7]
    - Ambulation yes#After play: 12.6 [10.1, 15.2]
- RMSSD (ms)
  - Time period
    - Baseline: 8.3 [6.8, 9.7]
    - Play: 8.9 [7.5, 10.4]
    - After play: 9.4 (7.9, 10.8]

**20 sec**

- HR (bpm)
  - Time period
    - Baseline: 147.7 [138.0, 157.3]
    - Play: 143.3 [133.7, 152.9]
    - After play: 143.6 [134.0, 153.3]
- SDNN (ms)
  - Time period
    - Baseline: 13.3 [10.1, 16.5]
    - Play: 11.0 [7.7, 14.2]
    - After play: 14.6 [11.4, 17.8]
- RMSSD (ms)
  - Time period
    - Baseline: 8.4 [6.0, 10.8]
    - Play: 7.9 [5.5, 10.3]
    - After play: 9.2 [6.8, 11.7]
